# The relationship between risk perceptions and negative emotions in the COVID-19: a meta-analysis

**DOI:** 10.3389/fpsyg.2024.1453111

**Published:** 2024-08-26

**Authors:** Ruihua Zhou, Kan Shi, Xudong Song, Shuqi Li, Wei Zhou

**Affiliations:** ^1^Department of Psychology, School of Education, Wenzhou University, Wenzhou, China; ^2^School of Public Administration and Policy, Renmin University of China, Beijing, China

**Keywords:** COVID-19, risk perception, negative emotions, tight and loose culture, meta-analysis

## Abstract

**Background:**

COVID-19, as a significant public health issue, has had a major impact on the mental health of people worldwide. Research shows a significant positive correlation between individuals’ risk perception levels and negative emotions during the outbreak of COVID-19. However, some studies also suggest that the relationship between the two is not significant. Therefore, we will conduct a meta-analysis to explore the relationship between risk perception and negative emotions from cultural, temporal, and individual psychological perspectives.

**Methods:**

Searches were conducted in the Web of Science, Pub Med, Google Scholar, PsycINFO, Scopus, and China National Knowledge Infrastructure databases, focusing on publications from January 2020 onwards, specifically targeting studies examining the relationship between risk perception and negative emotion during COVID-19.

**Results:**

A total of 58 papers with 85 effect sizes were meta-analyzed using Comprehensive Meta-Analysis 3.0 software, with a combined sample of 83,948 individuals. Risk perception of COVID-19 showed a moderate positive correlation with negative emotions (*r* = 0.211, 95%CI [0.18, 0.24]). There was no moderating effect of tight-loose cultures on the relationship between risk perception of COVID-19 and negative emotions. However, the epidemic period, gender ratio, and measurement methods did have moderating effects on the relationship between risk perception of COVID-19 and negative emotions.

**Conclusion:**

In future research, we can further develop theories related to the risk perception of COVID-19 and negative emotions, and based on these, formulate interventions to promote people’s mental health.

## Introduction

The outbreak of COVID-19 has caused profound harm to various parts of the world in the past 4 years, with significant negative impacts on physical and mental health. The WHO (2005) classified the coronavirus as an “international public health emergency” that poses a threat to human lives worldwide. The WHO still considers COVID-19 a public health emergency of international concern 3 years later (WHO, 2005). At the same time, due to the recurrent nature of the outbreak, the uncertainty of its spread and the strong transmissibility after mutation have led to a decrease in people’s psychological wellbeing (Talevi et al., 2020). The existing studies have found that in the context of the COVID-19 outbreak, negative emotions such as anxiety, depression, and sensitivity to social risks in the Chinese population increased, while life satisfaction and positive emotions decreased (Li et al., 2020; Rubin and Wessely, 2020).

Negative emotions refer to subjective experiences of unpleasantness and distress in response to adverse events in life. These emotions mainly include depression and anxiety, but also encompass feelings such as anger, fear, and discouragement (Parkitny and McAuley, 2010; Watson et al., 1988). COVID-19 has led to challenges in regulating these negative emotions, resulting in clinical symptoms like depression and anxiety (Wang C. et al., 2020). Thus, this study focuses on three types of emotions: (1) General negative emotions: characterized by unpleasant and distressing subjective experiences, including various emotions such as anxiety, depression, anger, and discouragement. (2) Anxiety: A tense emotional state experienced when individuals perceive potential dangers or threats in their current situation (Spielberger and Reheiser, 2009). (3) Depression: prolonged emotional states characterized by sadness and fear (Jackson, 2008).

Risk is defined by the magnitude of event probabilities and their consequences (Freudenburg, 1988). In the field of psychological research on risk, there is a primary focus on risk perception. Risk perception refers to an individual’s awareness and understanding of various objective risks in the external environment, emphasizing subjective feelings in this process (Slovic, 1987). Following Slovic and Peters (2006) definition, we define the risk perception of COVID-19 as an individual’s subjective evaluation and response to the potential risks and consequences associated with COVID-19-related information and situations.

Numerous studies have demonstrated a positive correlation between risk perceptions and negative emotions (Hogarth et al., 2011; Johnson and Tversky, 1983; Kopetz, 2017; Sjöberg, 2007; Yuen and Lee, 2003). This conclusion has also been confirmed in the context of COVID-19 (Barattucci et al., 2020). Research indicates that risk perception of COVID-19 is associated with higher levels of negative emotions (Han Q. et al., 2021). According to cognitive appraisal theory, individual emotions are determined by their appraisal of events. When assessing threats with high uncertainty, such as COVID-19, individuals are more likely to experience anxiety (Epstein, 1994; Lazarus, 1968; Lazarus, 1991). A study conducted during COVID-19 highlighted that worry can activate a dysfunctional overestimation of threats, leading to psychological distress. However, it can also activate functional appraisals, enhancing self-efficacy and thereby reducing distress (Diotaiuti et al., 2023). The transactional stress theory suggests that when individuals perceive an unavoidable health risk, they are more likely to use emotion-focused coping strategies (Lazarus and Folkman, 1984). In such cases, the relationship between risk perception of COVID-19 and negative emotions might be stronger (Chen et al., 2022). The social amplification framework hypothesizes that network communication will exaggerate the risks of COVID-19, causing people to focus more on negative information, thereby increasing negative emotions and risk perception (Ng et al., 2018). The behavioral immune system (BIS) theory suggests that when individuals face pathogen threats, the BIS triggers negative emotions such as disgust and anxiety, and enhances disease perception to avoid infection (Li et al., 2020; Makhanova and Shepherd, 2020; Neuberg et al., 2011). Furthermore, infectious diseases (such as COVID-19) have had a significant impact on human genetic evolution, making people more likely to overestimate related risks and experience negative emotions.

However, existing studies suggest that there may be moderating variables between risk perception and negative emotions. Tight-loose cultures, as a new cultural dimension, have their roots in ancient history and philosophy, and were first studied anthropologically by distinguishing between “tight” and “loose” traditional societies (Pelto, 1968). Tight cultures have high social norm strength and low tolerance for deviant behavior, while loose cultures are the opposite (Gelfand et al., 2011). A study highlighted that during the threat of COVID-19, governments in tightened cultural regions implemented stricter intervention policies to curb the spread of the disease, thereby protecting the population from the threat (Gelfand et al., 2011; Gelfand et al., 2021). Moreover, Dong et al. (2021) conducted a cross-sectional study that demonstrated cultural tightness–looseness as a moderating variable, relieving the positive correlation between risk perception and depression and anxiety. In the context of COVID-19, research on gender differences in risk perception has yielded various results (Jin et al., 2020; Dryhurst et al., 2022). Historical experiences suggest that the risks associated with COVID-19 may have a greater impact on females (for example, immune function in pregnant females is suppressed, leading to more severe viral damage to the body), resulting in higher risk perception among females and triggering more intense negative emotions (Ackerman et al., 2018). Additionally, some scholars argue that due to the high heterogeneity in the conceptualization of risk perception, research findings vary (Vieira et al., 2022). Currently, there are mainly two types of risk perception models. The first is from the Health Belief Model (HBM), which includes Perceived Severity (an individual’s awareness of the consequences of diseases), Perceived Susceptibility (an individual’s assessment of the likelihood of contracting a disease), and Perceived Vulnerability (an individual’s belief in their susceptibility to infection and consequent harm) (Brewer et al., 2007; Champion and Skinner, 2008). The second is based on Slovic’s paradigm, which primarily involves the dimensions of Familiarity and Controllability (Gan and Fu, 2022). Current research rarely discusses the distinctions between these two types of risk perception measurement models in the context of COVID-19, and it is still unknown whether they influence the relationship with negative emotions. Finally, research has shown that the relationship between COVID-19 risk perception and depression is influenced by the pandemic period (Liu et al., 2024). Dyer and Kolic (2020) found through semantic network analysis that the phenomenon of psychophysical numbing under COVID-19 is increasingly evident, demonstrating that negative emotions gradually decline over the duration of the pandemic, leading to a numbed mindset. It is uncertain whether this change affects the relationship between risk perception and negative emotions. Given that humanity may need to coexist with COVID-19 for an extended period, it is crucial to study the dynamic relationship between COVID-19 risk perception and negative emotions over different time periods.

Therefore, the purpose of this meta-analysis and systematic review is to explore the relationship between risk perception and negative emotions during COVID-19 and to investigate four potential moderating variables: cultural tightness–looseness, gender ratio, epidemic period, and risk perception measurement models.

## Methodology

This article has been reported by the Preferred Reporting Items for Systematic Reviews and Meta-Analyses (PRISMA) checklist.

### Data sources

All literature data are from the following databases: Web of Science, Pub Med, PsycINFO, Scopus and China National Knowledge Infrastructure. We used “risk perception COVID-19” AND “Negative affect” OR “Anxiety” OR “Depression” OR “Negative emotion” as the keywords to search in the PubMed dataset and used “TS = (risk perception COVID-19) AND ((TS = (Negative emotion)) OR (TS = (Anxiety)) OR (TS = (depression)))” in the Web of science dataset. We retrieved articles spanning from June to September 2022, totaling 1976 documents.

### Inclusion criteria and exclusion criteria

1. This study includes correlation coefficients and other metrics that can be converted into Fisher’s *Z-*values for meta-analysis, such as regression coefficients and chi-square values. Data that cannot be transformed in this manner will be excluded. (2) Negative emotions include “negative emotions,” “anxiety,” or “depression.” Considering that negative emotion measurements specific to events such as COVID-19 may temporarily exaggerate due to situational influences (Davis et al., 2011), we have chosen to exclude such specific measures (e.g., the COVID-19 Anxiety Scale: How anxious are you when discussing COVID-19?). (3) Participants in this study include all individuals affected by COVID-19, excluding those with mental disorders or severe illnesses, as well as COVID-19 patients, healthcare workers, or individuals with special experiences related to COVID-19. (4) This study will include empirical research but will not include review studies such as meta-analyses, scoping reviews, or systematic reviews. (5) The study must be published after January 2020, that is, after the outbreak of COVID-19. (6) Risk perception measurement methods include perceived severity, perceived possibility, perceived vulnerability, and the Slovic paradigm. Methods not falling into these categories are defined as “others.”

### Literature coding and quality evaluation

Each study was reviewed and independently coded by two psychology professionals who underwent a one-month training program and used a standardized template (Supplementary Table S2) for data extraction. Differences in coding were resolved through consultation, with the final coding determined by the corresponding author. The final encoding consistency is 100%. The coding content includes author information, publication year, male ratio, age, country, tight-loose cultures, epidemic period, and risk perception measurement method. For the coding of tightness culture, Fischer and Karl (2022) were adopted, combined with Gelfand et al. (2011, 2021)‘s measurements of national tightness. For countries or regions not included in the study, we did not perform coding. Due to standardization and centralization, A score of 0 represents the average level of tight-loose culture across countries. Scores above 0 indicate a tight culture, characterized by stronger social norms and lower tolerance for deviant behavior, while those below 0 were coded as loose cultures. Based on the Health Belief Model, risk perception is encoded as “perceived likelihood,” “perceived severity,” “perceived vulnerability” or a combination of these three measurement methods (Brewer et al., 2007; Champion and Skinner, 2008). According to Slovic’s psychological measurement model, familiarity and controllability are defined as the “Slovic paradigm.” If the measurement methods do not belong to the above, they are coded as “the others.” For effect size, linear regression *β-*values are converted into correlation coefficients using the formula of Peterson and Brown (2005). In this study, we used the Joanna Briggs Institute (JBI) critical appraisal checklist to assess the quality of cross-sectional studies (Munn et al., 2020). Studies with scores below 50% were excluded to ensure the reliability of the included research and the credibility of the results.

### Meta-analytic process

In the study, Comprehensive Meta-Analysis 3.0 software was used for the main effect test and the adjustment effect test. We conducted subgroup analysis on negative emotions, a method that divides a specific variable into different groups and examines differences between these groups to determine whether there are variations between different dependent variable indicators. If there are differences, they will be analyzed separately later. When there are multiple similar effect size indicators in a single study, the average effect size of multiple correlation coefficients is used. We will further explore the potential impact of non-independent effects on the results through sensitivity analysis. Specifically, we will exclude studies with non-independent effects to examine changes in the overall effect size.

Due to differences in research characteristics such as risk perception measurement methods and types of negative emotions, a random effects model was used to aggregate effect sizes. The model uses a weighted average to estimate the overall effect size, considering not only the within-study variance but also the between-study variance. On the tests of heterogeneity, the variability of the effect size in the main research was evaluated by the significance of *Q-*test results, *I^2^*, Tau^2^. An *I^2^ >* 75% indicates high heterogeneity, suggesting the appropriateness of the random effects model (Higgins et al., 2003).

Publication bias was tested qualitatively using funnel plot distribution of effect sizes. Quantitatively, Egger’s regression coefficient and Begg rank correlation test, with significant results indicating potential bias (Begg and Mazumdar, 1994; Sterne and Egger, 2001). Additionally, Orwin’s fail-safe N test and Classic Fail-safe N tests were used to determine how many unpublished studies are needed to make the overall effect size trivial or the *p*-value non-significant. Finally, the trim and fill method was used to correct for publication bias by adjusting for missing studies.

We employed subgroup analysis for categorical moderating variables, ensuring that at least three effect sizes were included at each level (Song et al., 2014). To test the effects of continuous moderating variables, this study employed a random effects model meta-regression.

## Results

### Literature inclusion and quality evaluation

**Figure 1:**
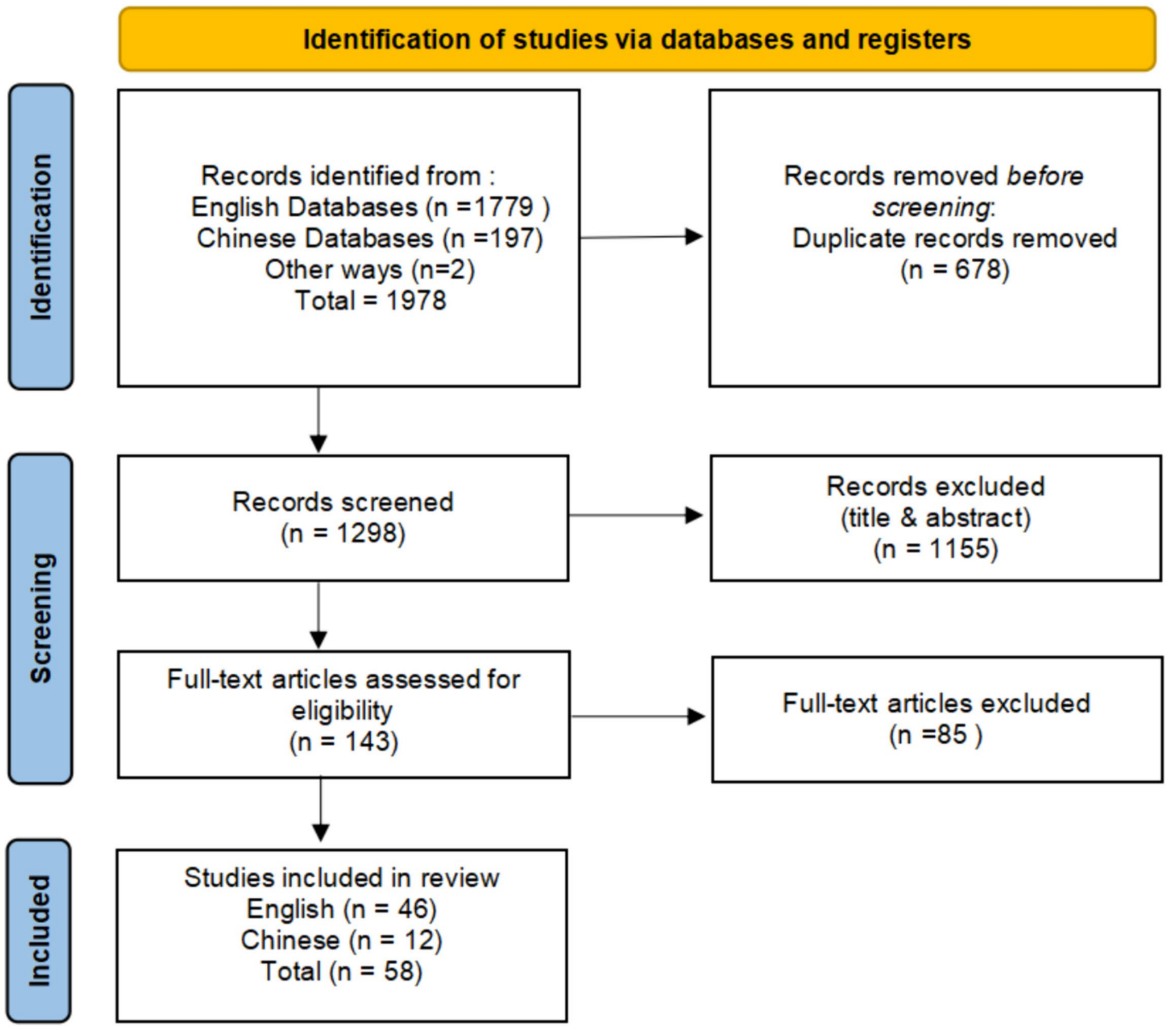
Process of selecting and screening articles.

According to the search strategy (Supplementary Table S1), a total of 58 studies were included in this meta-analysis, comprising 85 effect sizes and 83,948 subjects. This includes doctoral and master’s theses, with 12 studies in Chinese and 46 in English. Negative emotions include 19 effect size (*N* = 23,722), anxiety includes 41 effect size (*N* = 54,215), and depression includes 25 effect size (*N* = 40,592). Detailed data are shown in Supplementary Table S2. The present meta-analysis contained a total of 58 primary studies, 41 of which were from tight cultures. For data encoding, Cohen’s Kappa (k) scored by two raters is 0.89–0.94. Of the quality assessments, all the studies scored above 50% (Supplementary Table S3). The specific process is shown in Figure 1.

### Heterogeneity

The analysis results show that the overall *Q-*value is 2190.04, the *I^2^*-value is 96.16% and more than 75%, indicating a high level of heterogeneity in the results. Therefore, a random-effects model is appropriate (Higgins et al., 2003). And further explore other moderating variables that lead to heterogeneity of research results (Table 1).

**Table 1:** Heterogeneity test between risk perception of COVID-19 and negative emotion.

Indicator	k	*Q_B_*	*df*	*P*-value	*I^2^*	*Tau^2^*	*SE*	Variance	*Tau*
Negative emotion	19	1107.95	18	0.000	98.38	0.04	0.017	0.000	0.20
Anxiety	41	650.08	40	0.000	93.85	0.01	0.005	0.000	0.11
Depression	25	369.82	24	0.000	93.51	0.01	0.005	0.000	0.01
Overall indicators	85	2190.04	84	0.000	96.16	0.017	0.005	0.000	0.129

SE, standard error.

### Overall analyses

For the 58 papers analyzed, according to the analysis (as shown in Table 2), the overall effect size between risk perceptions and negative emotions of COVID-19 was *r* = 0.211 (*K* = 85) with 95%CI [0.18, 0.24] and no 0 in the interval, showing a moderate correlation, and the correlation coefficients for each sub-indicator of negative emotion, anxiety, and depression were 0.17 (*K* = 19, 95%CI [0.08, 0.25]), 0.24 (*K* = 41, 95%CI [0.21, 0.27]), 0.20 (*K* = 25, 95%CI [0.16, 0.24]). The data analysis conducted using subgroup analyses highlighted no significant differences between the groups regarding negative emotions (*Qb* = 3.78, *p* = 0.156), so the analysis of negative affect in the latter part was only for the overall indicators.

**Table 2:** Overall effect size between risk perception of COVID-19 and negative emotion.

Indicator	k	95%CI			two-tailed	
		*r*	Lower	Upper	*Z*	*P*
Negative emotion	19	0.17	0.08	0.25	3.68^***^	0.000
Anxiety	41	0.24	0.21	0.27	13.40^***^	0.000
Depression	25	0.20	0.16	0.24	9.55^***^	0.000
Overall indicators	85	0.21	0.18	0.24	14.63^***^	0.000

^*^p < 0.05, ^**^p < 0.01, ^***^p < 0.001.

### Sensitivity analysis

We performed the following sensitivity analyses to ensure the stability of our results. Firstly, we compared the overall effect sizes from the random effects model (*r* = 0.211) and the fixed effects model (*r* = 0.18). Secondly, we excluded studies using the average of multiple effect sizes (4 effect sizes were computed using average effect values, constituting 4.7% of the total studies). After exclusion, the overall effect size was 0.213, and the heterogeneity test showed that *I^2^* = 96.19%. Thirdly, using leave-one-out analysis, the results showed that the effect size *r*-value fluctuated between 0.206 and 0.216. The above sensitivity analysis results indicate that the overall results are relatively stable.

### Moderating effect

Firstly, for continuous moderating variables, random effects meta-regression was used, and the results showed that gender ratio moderated risk perceptions and negative emotions for COVID-19. The regression coefficient of the male ratio on the effect size was significant *β* = 0.46 (this indicates that the change of one male ratio unit leads to 0.46 times change of effect size), 95% CI [0.27, 0.65], *p* < 0.001, *R^2^* = 29%. Meanwhile, for the subgroup with k (this implies the existence of a pairwise relationship of correlation coefficients) <3. For example, since there were only two sets of correlation coefficients in 2020.09, they were not included in the subgroup analysis. Finally, for categorical moderating variables, subgroup analysis was taken, and the results showed (Table 3) that.

**Figure 2:**
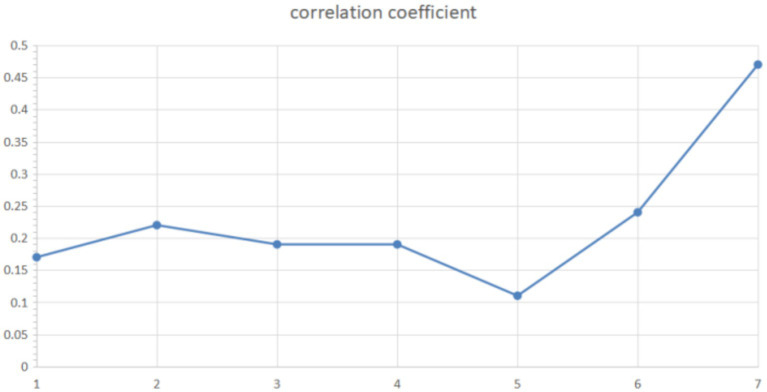
Epidemic period curve. The vertical axis represents the effect size, and the horizontal axis represents the epidemic period. Numbers 1–6 represent January to June 2020, while 7 represents 2021 and later.

1. There was no moderating effect of tight-loose cultures on risk perceptions and negative emotions of COVID-19, *QB* = 0.07, *p* = 0.785. For the insignificant moderating effect of tight-loose cultures, we conducted a further analysis by splitting tight culture and loose culture into two subgroups and observing the correlations under the corresponding cultures (Table 4). Excluding studies lacking corresponding cultural codes, we still did not find significant differences in the association between risk perceptions and negative emotions under tight-loose cultures. However, some numerical trends were found, for example, the association between risk perception and depression was slightly stronger in the tight culture (*r* = 0.221) than in the loose culture (*r* = 0.188), while the opposite was true for negative emotions (in the tight culture *r* = 0.159, in loose culture *r* = 0.212). The anxiety group showed a similar effect size (in tight culture *r* = 0.258, in loose culture *r* = 0.244). We also used the continuous variable of cultural tightness for supplementary meta-regression analysis. The regression coefficient was not significant *β* = −0.08, 95%CI [−0.17, 0.004], *p* > 0.05, *R^2^* = 1% (Table 5).
2. Risk perception measures moderated risk perception and negative emotions of COVID-19, *QB* = 18.22, *p* = 0.011, where the combination based on perceived severity and perceived possibility showed the strongest positive correlation (*r* = 0.28) between risk perceptions and negative emotions of COVID-19 (when other tools are excluded), while the combination based on Slovic’s familiarity, controllability showed the weakest positive correlation (*r* = 0.12).
3. The epidemic period moderated risk perceptions of COVID-19 and negative emotions, *QB* = 15.66, *p* = 0.016. Risk perception was most strongly positively associated (*r* = 0.47) with negative emotions in the time period 2021 and beyond, and least positively associated (*r* = 0.11) in the time period May 2020. In addition, we found an approximate inverted U curve of the effect sizes between January 2020 and May 2020 (although numerically this appears to be very weak), as shown in Figure 2. In addition, we also conducted a meta-regression analysis to test the moderating effect by using the epidemic period as a continuous variable. Specifically, we used the months since the outbreak of the pandemic as a continuous numerical variable (for example, January 2020 was 1, and February 2021 was 14). The regression coefficient was significant *β* = 0.02, 95%CI [0.01, 0.03], *p* < 0.01, *R^2^* = 2% (Table 5).

**Table 3:** Moderating effect between risk perception of COVID-19 and negative emotion.

Moderating effect	Heterogeneity test			Category	k	*r*	95%CI	
	*Q_B_*	*df*	*p*				Lower	Upper
TL	0.07	1	0.785	L	21	0.22	0.17	0.27
				T	55	0.22	0.18	0.25
Measurement	18.22	7	0.011	P	20	0.17	0.13	0.22
				S	10	0.18	0.12	0.25
				Slovic	11	0.12	0.00	0.23
				SP	12	0.28	0.20	0.35
				SV	7	0.16	0.1	0.22
				SVP	4	0.25	0.20	0.31
				V	8	0.21	0.15	0.26
				O	12	0.33	0.23	0.42
Epidemic Period	15.66	6	0.016	2020.01	3	0.17	−0.03	0.35
				2020.02	24	0.22	0.16	0.28
				2020.03	21	0.19	0.12	0.26
				2020.04	15	0.19	0.14	0.23
				2020.05	6	0.11	0.05	0.16
				2020.06	4	0.24	0.01	0.37
				2021 and later	4	0.47	0.24	0.64

L, Loose culture; T, tight culture; P, perceived possibility; S, perceived severity; Slovic, familiarity and controllability; SP, Combination of perceived possibility and perceived severity; SV, Combination of perceived severity and perceived vulnerability; SVP, Combination of perceived severity, perceived vulnerability and perceived possibility; V, perceived vulnerability; O, the others.

**Table 4:** Analysis of moderating effects of outcome variables.

Outcome	Heterogeneity test			Category	k	*r*	95%CI	
	*Q_B_*	*df*	*p*	Culture			Lower	Upper
A	0.126	1	0.722	T	24	0.258	0.211	0.304
				L	12	0.244	0.183	0.303
D	0.27	1	0.604	T	16	0.221	0.177	0.265
				L	6	0.188	0.065	0.305
N	0.688	1	0.407	T	16	0.159	0.135	0.287
				L	3	0.212	0.057	0.258

A, Anxiety; D, Depression; N, Negative emotion.

**Table 5:** Results of meta regression.

Moderators	k	*B*	95% CI	*P*	*R^2^*
Age	58	−0.0028	(−0.005, −0.000)	0.02	2%
Gender	84	0.46	(0.27, 0.65)	0.000	29%
epidemic period	79	0.02	(0.01, 0.03)	0.01	2%
T-L	75	−0.08	(−0.17,0.004)	0.064	1%

B, non-standardized regression coefficient; *R*^2^, proportion of variance explained.

### Publication bias

**Figure 3:**
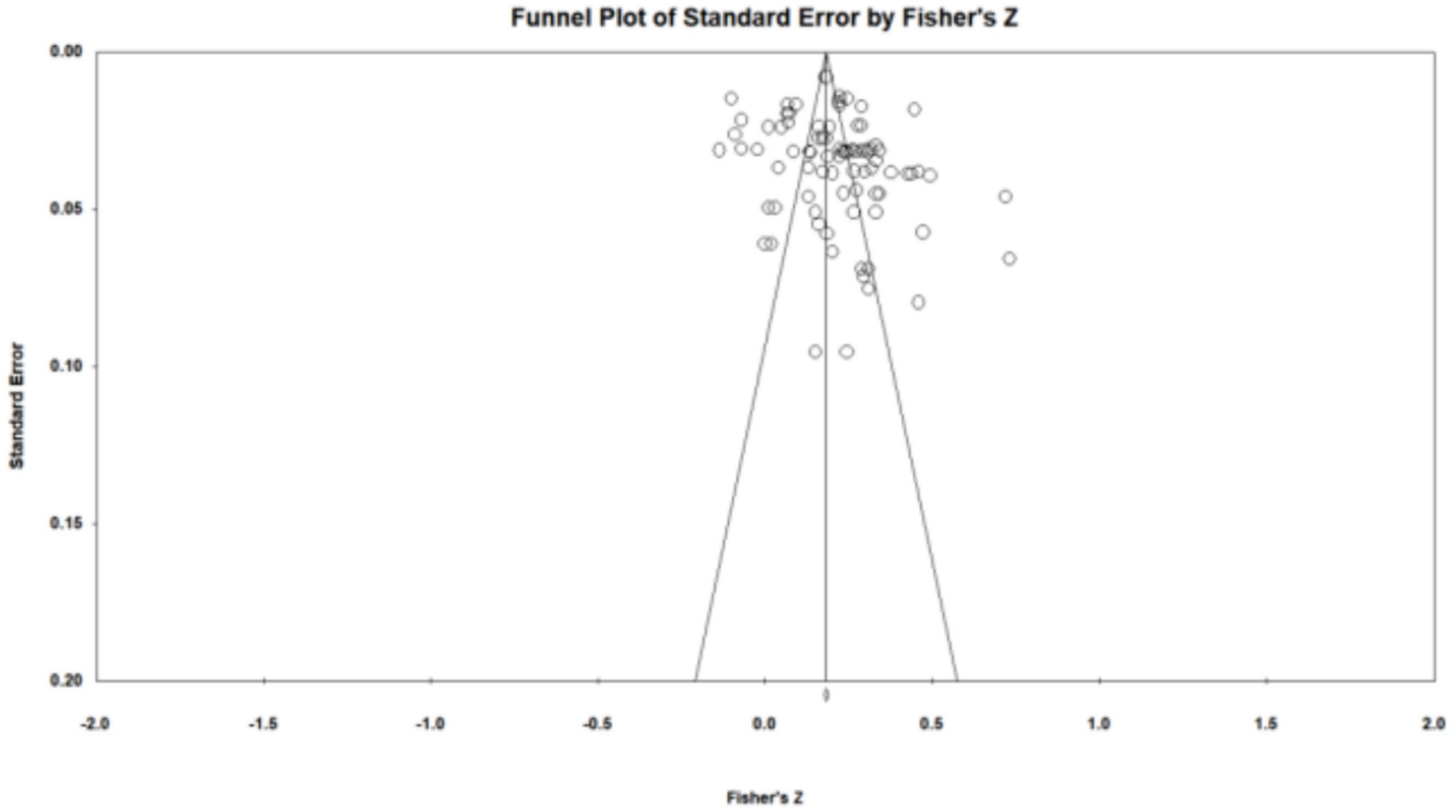
Funnel plot.

When there is a possibility of publication bias, effect sizes show an asymmetric distribution on the funnel plot (Borenstein et al., 2009). From the funnel chart observation (as shown in Figure 3), the effect sizes are mostly concentrated in the upper part of the funnel, and the distribution is uniform and symmetrical. The intercept of the Egger linear regression is 2.01, 95%CI [− 0.12, 4.15], and the *p*-value is 0.064. The *p*-value of the Begg test is 0.093. The results are not significant. The trim and fill test found 17 missing effect sizes on the left side of the funnel plot. These effect sizes were included in the analysis to get a new weighted effect size *r* = 0.161. The difference from the observed effect size is 23.7%, which belongs to the moderate cut-off value in publication bias (Chang et al., 2022). The classic fail-safe number is 4145, which is >5 K + 10 standard. The value of the classic fail-safe number indicates that at least 4,145 studies are needed to make the results insignificant. Orwin’s fail-safe *N-*value was 70, this means that when 70 articles with a correlation of 0.00 are included, the effect size will be lower than 0.1. Based on the above results, there is a low possibility of publication bias in this study. To further verify, we also used P-curve analysis, cumulative meta-analysis, and contour-enhanced funnel plot methods. The results also showed that there was a low probability of publication bias in the study (Supplementary Table S4).

## Discussion

### The relationship between risk perceptions and negative emotions of COVID-19

So far, this paper has examined the relationship between risk perceptions of COVID-19 and negative emotions through a meta-analysis, which includes 58 studies. The results reveal a moderate positive correlation (*r* = 0.211), with all three types of negative emotions strongly associated with risk perceptions. This aligns with the majority of research findings in the context of COVID-19 (Zhao et al., 2021), indicating that individuals with a heightened risk perception of COVID-19 also experience elevated levels of negative emotions. The moderate effect size indicated by the study may also be due to the numerous online measures that improved people’s mental health during COVID-19 (D’Oliveira et al., 2022). Furthermore, the lack of significant differences in the three types of negative emotions may be attributed to the simultaneous occurrence of depression and anxiety, which often coexist with considerable overlap in both psychological and clinical domains (Kessler et al., 2015). In terms of effect sizes, depression exhibits the lowest effect size, while anxiety shows the highest. This could possibly be attributed to a heightened awareness of unknown risks, such as anxiety, prompting individuals to promptly identify and avoid potential environmental cues associated with the virus. Another plausible explanation could be that, for an extended period, people have not felt helpless in the face of the virus spread. In summary, the results of the study indicate that the connection between risk perception and negative emotions remains significant in the context of COVID-19. The risk perception theories developed in previous pandemics are still applicable to the current situation of COVID-19.

### Tight-loose cultures

We found that the moderating effect of tight-loose culture on the risk perception of COVID-19 and negative emotions is not statistically significant. The reason for this may lie in the uneven distribution of tightness–looseness culture in the current sample (Tight culture = 55, Loose culture = 21). Numerous studies suggest that the moderating effect of tightness–looseness culture during the pandemic is significant (Tu et al., 2023). If the sample sizes from different cultures could be balanced, we speculate that a significant moderating effect of tightness–looseness culture might be observed. Another possible reason is that the studies included in this research focus on the early stages of COVID-19 or within the first 6 months after the outbreak. During this period, governments in regions with different cultures almost universally implemented various preventive measures, such as quarantine, social distancing, and the cancelation of large events (Rodríguez et al., 2022). This uniformity in response measures makes it difficult to reflect cultural differences in looseness–tightness.

### Gender ratio

Meta-regression analysis showed that a higher male ratio was associated with a stronger link between risk perceptions of COVID-19 and negative emotions. This finding contradicts the common observation that women are more prone to negative emotions and higher risk assessments (Campbell, 2013). One possible explanation is that men have higher mobility during the pandemic, which increases their exposure to the virus and subsequently elevates their levels of negative emotions (Semenova et al., 2021). Furthermore, men, as primary protectors and providers, are more likely to work outside. This increases their risk exposure and anxiety levels. Economic pressures and concerns about family stability may also contribute to higher levels of anxiety in men. Conversely, women’s heightened sensitivity to risk leads them to adopt more proactive measures to mitigate risks, potentially reducing the link between risk perceptions and negative emotions (Rana et al., 2021).

### Measurement method

The meta-analysis results indicated that, excluding other measures, the combination of perceived severity and possibility was the strongest predictor of the relationship between COVID-19 risk perceptions and negative emotions, while Slovic’s measurement paradigm was the weakest. One possible explanation is that familiarity and controllability measures lead people to judge risk more subjectively, whereas perceived possibility, severity, and vulnerability focus on objective, observable attributes of risk (Capone et al., 2021). Strong preventive measures and information about the virus and health behaviors at the pandemic’s outset enhanced people’s subjective motivation in risk perception, reducing the association with negative emotions. However, infection and mortality rates continued to rise during the pandemic’s initial phase (Baud et al., 2020). Therefore, objective epidemic risk still threatened people’s health security, and objective measures of perception remained strongly associated with negative emotions.

### Epidemic period

Meta-analysis showed that the correlation between risk perceptions and negative affect increased until March 2020, decreased after the WHO declared COVID-19 a global outbreak on March 11, and spiked again in June 2020, peaking in 2021. Studies have found that risk perceptions and anxiety levels were higher during the pandemic’s initial period, declining sharply over time (Savadori and Lauriola, 2022; Wang Z. et al., 2021; Qiao et al., 2023). According to the diffusion of innovation theory (Rogers et al., 2014), it takes time for people to absorb COVID-19 risk information, and as they receive more, their risk assessment increases. Early in the pandemic, limited information weakened people’s sense of control and heightened their sense of threat. Emotional information spreads quickly via social media, strengthening the link between risk perceptions and negative emotions (Brady et al., 2017).

In the later stages of the pandemic, meta-analytic results partially validate the phenomenon of psychological numbness. The high cost of maintaining intense negative emotions over time prompts people to start avoiding these emotions (Li et al., 2021). This may explain the gradual decrease in effect size. Risk perceptions were more strongly associated with negative emotions before the pandemic than after. This shift could be due to increased information availability, governmental control, and psychological adaptation (Tedaldi et al., 2022). The effect size spiked in June 2020 and June 2021, likely related to the coronavirus mutations. As people received more information about new variants, uncertainty rose, temporarily increasing the correlation between risk perceptions and negative emotions. Therefore, the effect values varied significantly across different periods, reflecting the epidemic’s impact on psychological states.

### Limitations

However, this study also has some shortcomings and perspectives: (1) As negative emotions contain many connotations and few studies have further sorted them out, the current study only includes the common indicators of mental health: negative emotions, depression, and anxiety. (2) It is necessary to discuss whether the participants included in the current study can adequately and effectively reveal the relationship between risk perception of COVID-19 and negative emotions. Studies have shown that more than one-third of healthcare workers experienced varying degrees of anxiety and depression during COVID-19 (Amin et al., 2020). Due to the widespread impact of COVID-19, it is necessary to include a broader range of populations in future studies. Additionally, it is important to explore whether the research results change over the duration of. For instance, people may gradually lower their risk assessment of the pandemic, which necessitates including more literature in future studies for further examination. (3) The moderating effect was not significant because the study of tight-loose cultures is still in its early stages, and each country experiences dynamic changes in its degree of tightness and looseness. This indicates the need for longitudinal studies on tight-loose cultures over time. For the epidemic period, our method of dividing by month needs improvement. Future research should use more refined coding or other methods. Regarding risk perception measurement, while mainstream models were used, other studies suggest that risk perceptions should include more questions and employ more accurate methods. Future research should integrate newly developed risk perception of COVID-19 measurements. (4) This study focused on the relationship between risk perceptions of COVID-19 and negative emotions. Future research should compare these results with other epidemic periods to determine if the findings are specific to COVID-19 and to establish a comprehensive theoretical framework for risk perception under different epidemics. (5) Finally, the choice of research methods may introduce potential biases. Compared to the traditional bivariate meta-analysis used in this study, multilevel meta-analysis has been widely adopted in recent years due to its ability to better control for biases. Future research could consider employing more advanced statistical methods to reduce bias.

## Conclusion

In the research, we discuss the relationship between risk perceptions and negative emotions. Cognitive theory and evolutionary psychology theory have also appropriately predicted their results, which not only expands psychological research in the context of COVID-19, but also provides an integrated model for risk perception for future research. In a large number of COVID-19 research, this study used the meta-analysis method for the first time to explore the relationship between risk perceptions of COVID-19 and negative emotions. Firstly, it reconfirms the association between risk perceptions of COVID-19 and negative emotions. In addition, it also enriched the research on risk perception during COVID-19, and explored the influence of cultural factors, demographic factors, psychometrics and psychological factors. Thus, we provide empirical support for the field of mental health of the general population with a targeted and scientific basis and lay the foundation for future risk perception research and mental health intervention development.

## Data Availability

The datasets generated during and/or analysed during the current study are available from the corresponding author on reasonable request.
