# Supplementary material for: The relationship between risk perceptions and negative emotions in the COVID-19: a meta-analysis
Source: Front Psychol. 2024 Aug 26;15:1453111. doi: 10.3389/fpsyg.2024.1453111 (PMC11381260; doi:10.3389/fpsyg.2024.1453111)
Supplement: Supplementary file 1 [file Table_1.DOCX]

**PubMed：**

(risk perception COVID-19) AND ((Negative affect) OR (Anxiety) OR (depression) OR (Negative emotion))

**Web of science：**

TS=(risk perception COVID-19) AND ((TS=(Negative emotion)) OR (TS=(Anxiety)) OR (TS=(depression)))
