# Supplementary material for: The relationship between risk perceptions and negative emotions in the COVID-19: a meta-analysis
Source: Front Psychol. 2024 Aug 26;15:1453111. doi: 10.3389/fpsyg.2024.1453111 (PMC11381260; doi:10.3389/fpsyg.2024.1453111)
Supplement: Supplementary file 2 [file Table_2.DOCX]

**Supplementary Table1 Characteristics included in the meta-analysis study**

| Study | Year | Culture | T-L score | Correlation | Sample | Male ratio | Period | Measurement | Negative emotions | age | Country/region | Negative emotions scale |
| --- | --- | --- | --- | --- | --- | --- | --- | --- | --- | --- | --- | --- |
| Barattucci et al. | 2020 | L | −0.07 | 0.263 | 998 | 0.26 | 3 | S | N | 38.34 | ITA | Positive and Negative Affect Schedule |
|  |  | L | −0.07 | 0.237 | 998 | 0.26 | 3 | V | N | 38.34 | ITA | Positive and Negative Affect Schedule |
|  |  | L | −0.07 | 0.227 | 998 | 0.26 | 2 | S | A | 38.34 | ITA | State Anxiety Scale |
|  |  | L | −0.07 | 0.228 | 998 | 0.26 | 2 | V | A | 38.34 | ITA | State Anxiety Scale |
| Chen et al. and La Rosa | 2020 | T | 0.41 | 0.3 | 992 | 0.472 | 3 | SLO | D | 19.45 | CHN | Mental and behavioral questionnaire |
|  |  | T | 0.41 | 0.24 | 992 | 0.472 | 3 | P | D | 19.45 | CHN | Mental and behavioral questionnaire |
| Commodari | 2020 | L | −0.07 | 0.135 | 978 | 0.347 | 4 | S | N |  | ITA | Positive and Negative Affect Schedule |
| Dong et al. | 2020 | T | 0.41 | 0.27 | 1827 | 0.467 | 3 | V | A | 18.16 | CHN | GAD-7 |
|  |  | T | 0.41 | 0.28 | 1827 | 0.467 | 3 | V | D | 18.16 | CHN | PHQ-9 |
| Feng et al. | 2020 | T | 0.41 | 0.18 | 1346 | 0.27 | 2 | P | D | 19.76 | CHN | PHQ-9 |
|  |  | T | 0.41 | 0.17 | 1346 | 0.27 | 2 | P | A | 19.76 | CHN | GAD-7 |
|  |  | T | 0.41 | 0.16 | 1346 | 0.27 | 2 | P | N | 19.76 | CHN | Positive and Negative Affect Scale |
| Fu and Wang | 2022 | T | 0.41 | 0.267 | 522 | 0.372 | 21 | O | A | 21.77 | CHN | GAD-7 |
| Gan et al. | 2021 | T | 0.41 | -0.09 | 1452 | 0.138 | 3 | SLO | N | 33.5 | CHN | Positive and Negative Affect Scale |
| Gan and Fu | 2022 | T | 0.41 | -0.136 | 1038 | 0.266 | 3 | SLO | N | 20.5 | CHN | Positive and Negative Affect Scale |
| Germani et al. | 2020 | L | −0.07 | 0.255 | 1045 | 0.3 | 3 | O | A | 24.18 | ITA | State Anxiety Scale |
| Gu et al. | 2022 | T | 0.41 | 0.308 | 735 | 0.464 | 4 | SLO | N |  | CHN | Negative Emotions scale |
| Haliwa et al. | 2021 | L | −0.23 | 0.2 | 251 | 0.183 | 3 | O | A | 19.62 | USA | Depression Anxiety Stress Scale |
| Han et al. | 2021 | T | 0.78 | 0.01 | 413 | 0.346 | 5 | P | D | 69.09 | KOR | Geriatric Depression Scale |
|  |  | T | 0.78 | 0.03 | 413 | 0.346 | 5 | P | A | 69.09 | KOR | Geriatric Anxiety Inventory |
| Hu et al. | 2022 | T | 0.41 | 0.22 | 3443 | 0.356 |  | P | D | 26.25 | CHN | Symptom Checklist-90-Revised |
| Hubbard et al. | 2020 |  |  | 0.132 | 1006 | 0.386 | 6 | SV | A | 53 | SCO | PHQ-4 |
|  |  |  |  | 0.085 | 1006 | 0.386 | 6 | SV | D | 53 | SCO | PHQ-4 |
| Jaspal and Breakwell | 2022 | L | −0.23 | 0.28 | 214 | 0.238 | 9 | P | D |  | GBR | Center for Epidemiologic Studies Depression Scale |
|  |  | L | −0.23 | 0.3 | 214 | 0.238 | 9 | P | A |  | GBR | Generalized Anxiety Disorder Assessment |
| Lee et al. | 2021 | T |  | 0.3 | 180 | 0.3 | 2 | PV | A | 23.4 | TWN | State Anxiety Scale |
| Li et al. | 2020 | T | 0.41 | 0.24 | 4607 | 0.275 | 2 | S | N | 23.71 | CHN | negative emotion scale |
|  |  | T | 0.41 | -0.1 | 4607 | 0.275 | 2 | SLO | N | 23.71 | CHN | negative emotion scale |
| Li et al. | 2021 | T | 0.41 | 0.42 | 3037 | 0.764 | 2 | SP | N | 16.11 | CHN | Positive and Negative Affect Schedule |
| Li and Lyu | 2021 | T | 0.41 | 0.29 | 693 | 0.38 | 2 | S | A |  | CHN | GAD-7 |
|  |  | T | 0.41 | 0.17 | 693 | 0.38 | 2 | S | D |  | CHN | CES-D-10 |
| 1. Liu et al. | 2020 | T | 0.41 | 0.22 | 4991 | 0.496 | 2 | O | A |  | CHN | Zung Self-Rating Anxiety Scale |
| 1. Liu et al. | 2020 | T | 0.41 | 0.457 | 653 | 0.387 | 3 | SP | N |  | CHN | Negative Emotions scale |
| Luo et al. | 2022 | T | 0.41 | 0.32 | 1137 | 0.573 | 6 | SP | D |  | CHN | PHQ-2 |
| Malesza and Kaczmarek | 2021 |  | −0.51 | 0.33 | 1019 | 0.433 | 3 | P | A | 38.54 | POL | Anxiety Sensitivity Index-3 |
| Olagoke et al. | 2020 | L | −0.23 | 0.23 | 501 | 0.447 | 3 | V | D | 32.44 | USA | PHQ-2 |
| Padmanabhanunni and Pretorius | 2021 |  |  | 0.16 | 337 | 0.228 | 3 | P | A | 21.95 | ZAF | State-Trait Anxiety Inventory |
|  |  |  |  | 0.16 | 337 | 0.228 | 3 | P | D | 21.95 | ZAF | CES-D |
| Peleg et al. | 2021 | L | −1.12 | 0.32 | 498 | 0.472 | 4 | S | A | 40.61 | ISR | State-Trait Anxiety Inventory |
|  |  | L | −1.12 | 0.33 | 498 | 0.472 | 4 | P | A | 40.61 | ISR | State-Trait Anxiety Inventory |
| Pramukti et al. | 2020 |  |  | 0.07 | 1985 | 0.2 | 4 | V | A |  |  | State-Trait Anxiety Inventory |
| Riesel et al. | 2021 | T | 0.18 | 0.24 | 113 | 0.372 | 2 | SP | D | 33.47 | DEU | Beck Depression Inventory–II |
|  |  | T | 0.18 | 0.15 | 113 | 0.372 | 2 | SP | A | 33.47 | DEU | State-Trait Anxiety Inventory |
| Rodrigues et al. | 2022 |  |  | 0.43 | 161 | 0.484 | 6 | O | A | 33.94 |  | GAD-7 |
| Rosi et al. | 2021 | L | −0.07 | 0.01 | 1765 | 0.265 | 4 | S | D |  | ITA | Profile of Mood States |
|  |  | L | −0.07 | 0.05 | 1765 | 0.265 | 4 | S | A |  | ITA | Profile of Mood States |
|  |  | L | −0.07 | 0.16 | 1765 | 0.265 | 4 | V | D |  | ITA | Profile of Mood States |
|  |  | L | −0.07 | 0.19 | 1765 | 0.265 | 4 | V | A |  | ITA | Profile of Mood States |
| Rubaltelli et al. | 2020 | L | −0.07 | 0.29 | 1031 | 0.32 | 3 | P | A | 30.79 | ITA | State-Trait Anxiety Inventory |
| Salazar et al. | 2021 | L | −0.60 | 0.403 | 677 | 0.499 | 4 | O | D | 48.75 | ESP | Depression Anxiety Stress Scale-21 |
|  |  | L | −0.60 | 0.412 | 677 | 0.499 | 4 | O | A | 48.75 | ESP | Depression Anxiety Stress Scale-21 |
| Shi et al. | 2021 | T | 0.41 | 0.072 | 2651 | 0.215 | 2 | P | A | 35.91 | CHN | Hospital Anxiety and Depression Scale |
|  |  | T | 0.41 | 0.068 | 2651 | 0.215 | 2 | P | D | 35.91 | CHN | Hospital Anxiety and Depression Scale |
| Sica et al. | 2021 | L | −0.07 | 0.04 | 742 | 0.27 | 3 | SP | D | 30.7 | ITA | Depression Anxiety Stress Scale-21 |
|  |  | L | −0.07 | 0.13 | 742 | 0.27 | 3 | SP | A | 30.7 | ITA | Depression Anxiety Stress Scale-21 |
| Tagin et al. | 2021 | T | −0.07 | 0.186 | 911 | 0.233 | 5 | SV | D | 41.61 | ITA | PHQ-9 |
|  |  | T | −0.07 | 0.219 | 911 | 0.233 | 5 | SV | A | 41.61 | ITA | GAD-7 |
| Torrente et al. | 2021 |  |  | 0.068 | 3617 | 0.145 | 5 | SV | D | 47.31 | ARG | PHQ-9 |
|  |  |  |  | 0.092 | 3617 | 0.145 | 5 | SV | A | 47.31 | ARG | GAD-7 |
| Wang, Rao and Han | 2021 | T | 0.41 | 0.286 | 200 | 0.545 | 21 | P | A | 27.87 | CHN | Self-Rating Anxiety Scale |
| Wang et al. | 2022 | T | 0.41 | 0.26 | 390 | 0.59 | 3 | O | A |  | CHN | Anxiety scale by Daniels et al (1997)^[1]^ |
|  |  | T | 0.41 | 0.15 | 390 | 0.59 | 3 | P | A |  | CHN | Anxiety scale by Daniels et al (1997)^[1]^ |
| Wu et al. | 2021 | T | 0.41 | 0.26 | 701 | 0.378 | 2 | SVP | D |  | CHN | CES-D |
| Xin et al. | 2022 | T | 0.41 | 0.183 | 304 | 0.424 | 2 | O | N |  | CHN | Positive and Negative Affect Schedule |
| Xu and Yan | 2022 | T | 0.41 | 0.32 | 847 | 0.412 |  | SP | A | 29.1 | CHN | State Anxiety Inventory |
| Yue et al. | 2020 | T | 0.41 | 0.44 | 308 | 0 | 2 | SLO | A | 31.63 | CHN | Self-Rating Anxiety Scale |
| Zhang | 2022 | T |  | 0.32 | 390 | 0.305 |  | SP | N | 33.28 | CHN | Positive and Negative Affect Schedule |
| Zhang et al. | 2021 | T |  | 0.02 | 272 | 0.511 | 4 | P | D | 70.63 | CHN | Depression Anxiety Stress Scales |
|  |  | T |  | 0 | 272 | 0.511 | 4 | P | A | 70.63 | CHN | Depression Anxiety Stress Scales |
| Zhao, Shi et al. | 2021 | T |  | 0.36 | 684 | 0.443 | 2 | SV | A | 22.43 | CHN | Anxiety of Negative emotion scale |
| Zhao, Ye and Ma | 2021 | T |  | 0.28 | 3341 | 0.338 | 2 | SLO | A | 19.57 | CHN | Self-Rating Anxiety Scale |
| Documents from CNKI | | | | | | | | | | | |  |
| An et al. | 2022 | T |  | 0.617 | 475 | 0.587 | 21 | O | A |  | CHN | Self-Rating Anxiety Scale |
| Chen | 2021 | T |  | 0.218 | 4096 | 0.396 |  | SLO | N |  | CHN | Emotional Health Response Scale |
| Hu et al. | 2020 | T |  | 0.304 | 1063 | 0.279 | 1 | SP | N | 26.89 | CHN | Negative Emotions Questionnaire for Public Health Events |
|  |  | T |  | 0.22 | 1063 | 0.279 | 1 | SP | D | 26.89 | CHN | Negative Emotions Questionnaire for Public Health Events |
| Jiang and Ma | 2022 | T |  | 0.2 | 679 | 0.604 | 2 | SLO | N |  | CHN | Positive and Negative Affect Schedule |
| Shi et al. | 2021 | T |  | -0.07 | 2144 |  | 3 | SLO | N |  | CHN | Depression Anxiety Stress Scale-21 |
| Wang, Chen et al. | 2021 | T |  | 0.28 | 1021 | 0.46 | 2 | SP | A | 26.94 | CHN | PROMIS |
| Wang, Li and Lu | 2020 | T |  | 0.43 | 698 | 0.38 |  | SVP | A |  | CHN | GAD-7 |
| Xu | 2022 | T |  | 0.624 | 235 | 0.332 | 21 | O | D |  | CHN | Self-Rating Depression Scale |
| Xu et al. | 2022 | T |  | -0.07 | 1071 | 0.392 |  | SLO | N |  | CHN | Depression Anxiety Stress Scale-21 |
| Ye et al. | 2021 | T |  | 0.13 | 477 | 0.256 | 2 | S | N |  | CHN | Positive and Negative Affect Schedule |
| Zhen and Zhou | 2020 | T |  | -0.023 | 1050 | 0.344 | 1 | O | A |  | CHN | DSM-5 Generalized Anxiety Assessment Program |
| Zhu et al. | 2022 | T |  | 0.18 | 15936 | 0.42 | 4 | SVP | D | 20.47 | CHN | PHQ-9 |
|  |  | T |  | 0.181 | 15936 | 0.42 | 4 | SVP | A | 20.47 | CHN | GAD-7 |

L=Loose culture ; T=tight culture

P=perceived possibility ; S=perceived severity ; Slovic=familiarity and controllability ; SP=Combination of perceived possibility and perceived severity ; SV=Combination of perceived severity and perceived vulnerability ; SVP=Combination of perceived severity, perceived vulnerability and perceived possibility ; V=perceived vulnerability; O=other tools

1=January 2020 ; 2=February 2020 ; 3=March 2020 ; 4=April 2020 ; 5=May 2020 ; 6=June 2020 ; 21=2021 and later

A=anxiety ; D=depression ; N=Negative emotions

[1]Daniels, K., Brough, P., Guppy, A., Peters‐Bean, K. M., & Weatherstone, L. (1997). A note on a modification to Warr's measures of affective well‐being at work. *Journal of Occupational and Organizational Psychology, 70*(2), 129-138.
