# Supplementary material for: The relationship between risk perceptions and negative emotions in the COVID-19: a meta-analysis
Source: Front Psychol. 2024 Aug 26;15:1453111. doi: 10.3389/fpsyg.2024.1453111 (PMC11381260; doi:10.3389/fpsyg.2024.1453111)
Supplement: Supplementary file 3 [file Table_3.DOCX]

| Studies | Clear eligibility criteria | Description of subject and setting | Valid & reliable method for exposure assessment | Standard criteria used for measurement | Confounding factors identification | Develop strategy for confounding factors | Valid & reliable method to measured outcomes | Appropriate statistical analysis | Total score out of 8 |
| --- | --- | --- | --- | --- | --- | --- | --- | --- | --- |
| Barattucci et al. | Yes | Yes | Yes | Yes | Yes | Yes | Yes | Yes | 8 |
| Chen et al. and La Rosa | Yes | Yes | Yes | Yes | No | No | Yes | Yes | 6 |
| Commodari & La Rosa | Yes | Yes | Yes | Yes | Yes | No | Yes | Yes | 7 |
| Dong et al. | Yes | Yes | Yes | Yes | Yes | Yes | Yes | Yes | 8 |
| Feng et al. | Yes | Yes | Yes | Yes | Yes | Yes | Yes | Yes | 8 |
| Gan et al. | Yes | Yes | Yes | Yes | Yes | Yes | Yes | Yes | 8 |
| Gan and Fu | Yes | Yes | Yes | Yes | Yes | No | Yes | Yes | 7 |
| Germani et al. | Yes | Yes | Yes | Yes | No | No | Yes | Yes | 6 |
| Gu et al. | Yes | Yes | Yes | Yes | Yes | No | Yes | Yes | 7 |
| Haliwa et al. | Yes | Yes | Yes | Yes | Yes | No | Yes | Yes | 7 |
| Han et al. | Yes | Yes | Yes | Yes | Yes | Yes | Yes | Yes | 8 |
| Hu et al. | Yes | Yes | Yes | Yes | Yes | No | Yes | Yes | 7 |
| Hubbard et al. | Yes | Yes | Yes | Yes | Yes | No | Yes | Yes | 7 |
| Jaspal and Breakwell | Yes | Yes | Yes | Yes | Yes | No | Yes | Yes | 7 |
| Lee et al. | Yes | Yes | Yes | Yes | Yes | No | Yes | Yes | 7 |
| Li & Yang et al. | Yes | Yes | Yes | Yes | Yes | Yes | Yes | Yes | 8 |
| Li & Luo et al. | Yes | Yes | Yes | Yes | Yes | No | Yes | Yes | 7 |
| Li and Lyu | Yes | Yes | Yes | Yes | Yes | Yes | Yes | Yes | 8 |
| 1. Liu et al. | Yes | Yes | Yes | Yes | Yes | No | Yes | Yes | 7 |
| 1. Liu et al. | Yes | Yes | Yes | Yes | Yes | No | Yes | Yes | 7 |
| Luo et al. | Yes | Yes | Yes | Yes | No | No | Yes | Yes | 6 |
| Malesza and Kaczmarek | Yes | Yes | Yes | Yes | Yes | No | Yes | Yes | 7 |
| Olagoke et al. | Yes | Yes | Yes | Yes | Yes | Yes | Yes | Yes | 8 |
| Padmanabhanunni and Pretorius | Yes | Yes | Yes | Yes | Yes | No | Yes | Yes | 7 |
| Peleg et al. | Yes | Yes | Yes | Yes | No | No | Yes | Yes | 6 |
| Pramukti et al. | Yes | Yes | Yes | Yes | No | No | Yes | Yes | 6 |
| Riesel et al. | Yes | Yes | Yes | Yes | No | No | Yes | Yes | 6 |
| Rodrigues et al. | Yes | Yes | Yes | Yes | Yes | No | Yes | Yes | 7 |
| Rosi et al. | Yes | Yes | Yes | Yes | Yes | Yes | Yes | Yes | 8 |
| Rubaltelli et al. | Yes | Yes | Yes | Yes | Yes | Yes | Yes | Yes | 8 |
| Salazar et al. | Yes | Yes | Yes | Yes | Yes | Yes | Yes | Yes | 8 |
| Shi et al. | Yes | Yes | Yes | Yes | Yes | No | Yes | Yes | 7 |
| Sica et al. | Yes | Yes | Yes | Yes | Yes | No | Yes | Yes | 7 |
| Tagin et al. | Yes | Yes | Yes | Yes | Yes | No | Yes | Yes | 7 |
| Torrente et al. | Yes | Yes | Yes | Yes | Yes | Yes | Yes | Yes | 8 |
| Wang, Rao and Han | Yes | Yes | Yes | Yes | Yes | No | Yes | Yes | 7 |
| Wang & He et al. | Yes | Yes | Yes | Yes | Yes | Yes | Yes | Yes | 8 |
| Wu et al. | Yes | Yes | Yes | Yes | No | No | Yes | Yes | 6 |
| Xin et al. | Yes | Yes | Yes | Yes | No | No | Yes | Yes | 6 |
| Xu and Yan | Yes | Yes | Yes | Yes | Yes | Yes | Yes | Yes | 8 |
| Yue et al. | Yes | Yes | Yes | Yes | Yes | No | Yes | Yes | 7 |
| Zhang | Yes | Yes | Yes | Yes | Yes | Yes | Yes | Yes | 8 |
| Zhang et al. | Yes | Yes | Yes | Yes | Yes | No | Yes | Yes | 7 |
| Zhao, Shi et al. | Yes | Yes | Yes | Yes | No | No | Yes | Yes | 6 |
| Zhao, Ye and Ma | Yes | Yes | Yes | Yes | No | No | Yes | Yes | 6 |
| **Documents from CNKI** | | | | | | | | | |
| An et al. | Yes | Yes | Yes | Yes | No | No | Yes | Yes | 6 |
| Chen | Yes | Yes | Yes | Yes | Yes | No | Yes | Yes | 7 |
| Hu et al. | Yes | Yes | Yes | Yes | No | No | Yes | Yes | 6 |
| Jiang and Ma | Yes | Yes | Yes | Yes | Yes | No | Yes | Yes | 7 |
| Shi et al. | Yes | Yes | Yes | Yes | No | No | Yes | Yes | 6 |
| Wang, Chen et al. | Yes | Yes | Yes | Yes | No | No | Yes | Yes | 6 |
| Wang, Li and Lu | Yes | Yes | Yes | Yes | No | No | Yes | Yes | 6 |
| Xu | Yes | Yes | Yes | Yes | Yes | No | Yes | Yes | 7 |
| Xu et al. | Yes | Yes | Yes | Yes | No | No | Yes | Yes | 6 |
| Ye et al. | Yes | Yes | Yes | Yes | Yes | No | Yes | Yes | 7 |
| Zhen and Zhou | Yes | Yes | Yes | Yes | Yes | Yes | Yes | Yes | 8 |
| Zhu et al. | Yes | Yes | Yes | Yes | Yes | No | Yes | Yes | 7 |
