# Supplementary material for: The relationship between risk perceptions and negative emotions in the COVID-19: a meta-analysis
Source: Front Psychol. 2024 Aug 26;15:1453111. doi: 10.3389/fpsyg.2024.1453111 (PMC11381260; doi:10.3389/fpsyg.2024.1453111)
Supplement: Supplementary file 4 [file Table_4.DOCX]

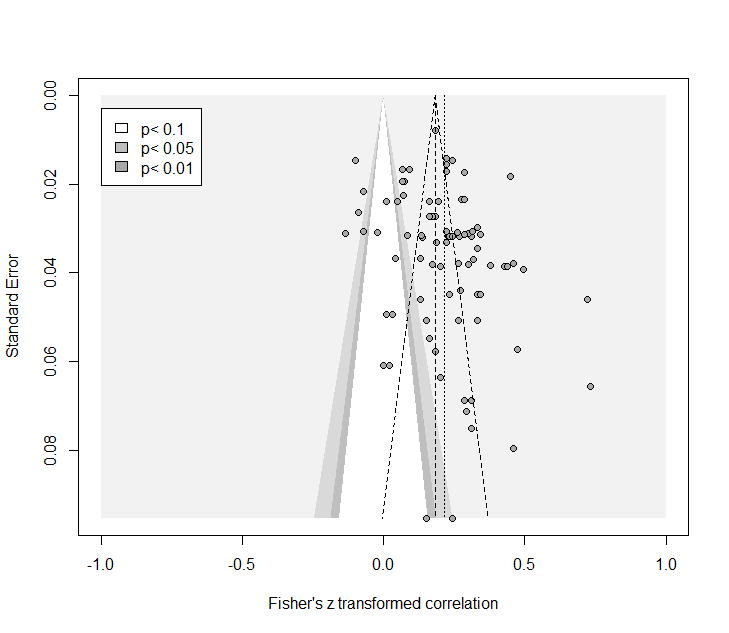


**Contour-enhanced funnel plot**

The different colored regions represent different p-values. Overall, the research showed a slight asymmetry, with 7 studies falling in the non-significant region.


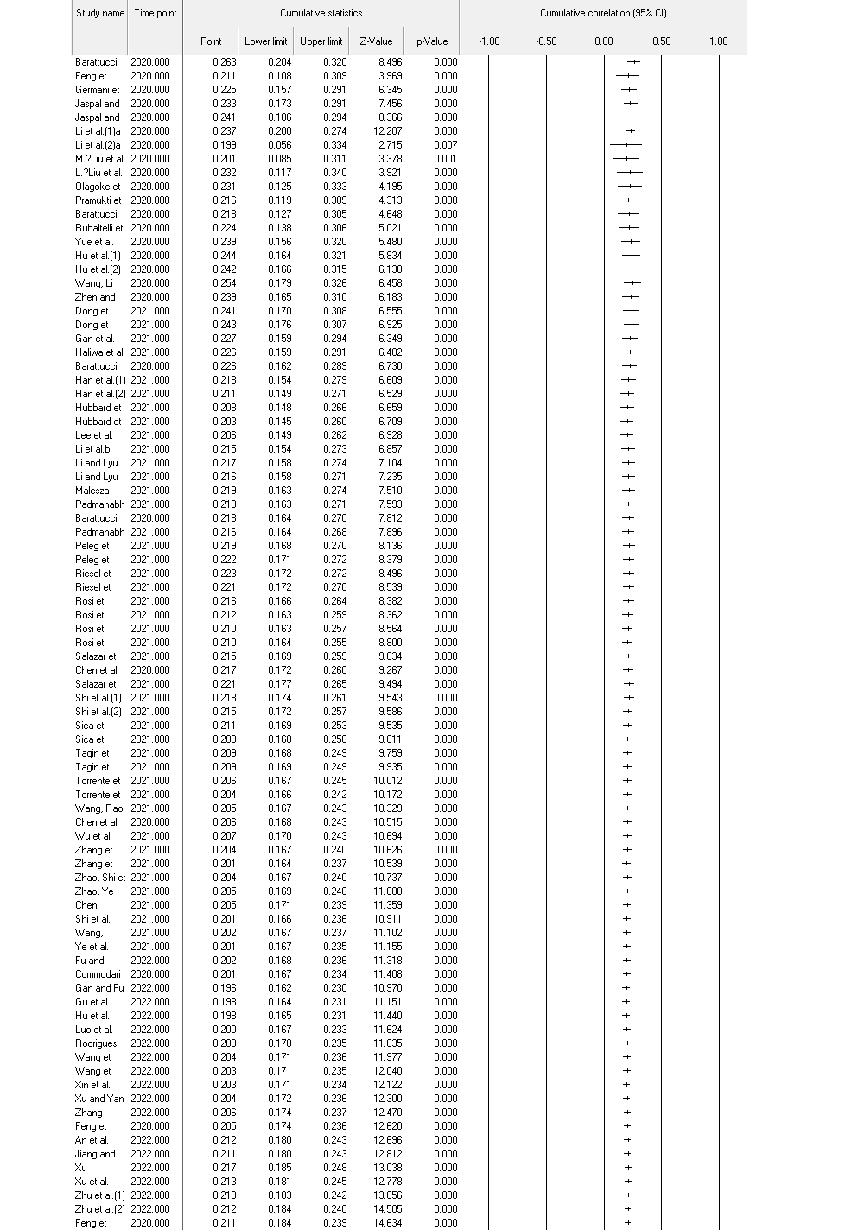


**Cumulative meta-analysis**


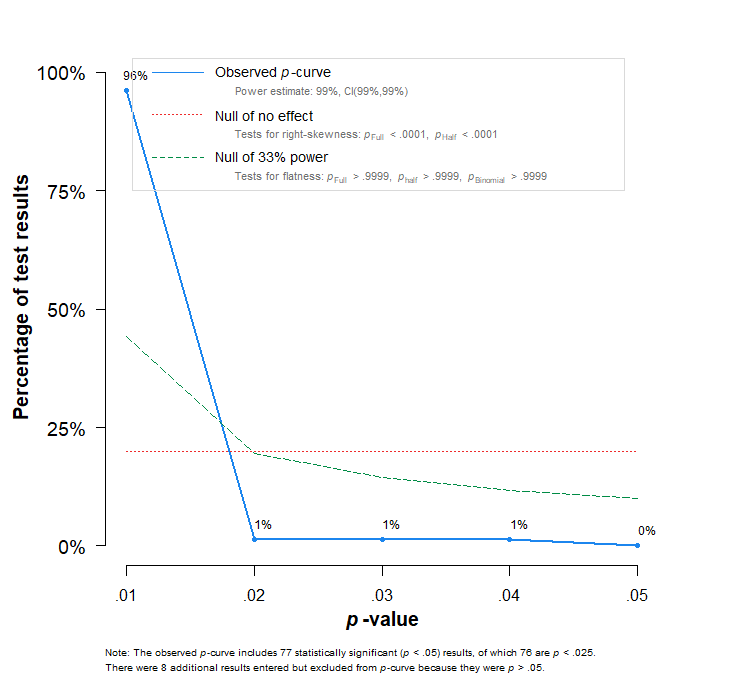


**P-curve analysis**

P-curve analysis showed significant results for three tests for rightskewness (p_Binomial_=1, p_Full_=1, p_Half_=1) and no significance for flatness tests (p_Binomial_ = 1, p_Full_ = 1, p_Half_ = 1).
